# Effect of Covid-19-pandemic on loneliness, well-being, significant others – Results of a national survey of adults in Hungary

**DOI:** 10.1192/j.eurpsy.2022.1351

**Published:** 2022-09-01

**Authors:** Z. Brys, F. Albert

**Affiliations:** 1 Semmelweis University, Mental Health Sciences, Budapest, Hungary; 2 Hungarian Academy of Sciences Centre of Excellence, Eötvös Loránd Research Network, Institute Of Sociology, Centre For Social Sciences, Budapest, Hungary

**Keywords:** Loneliness, Covid-19, mental health, significant others

## Abstract

**Introduction:**

Covid-19-pandemic is likely to have a substantial and long-term effect on the mental health of the adult Hungarian population.

**Objectives:**

To investigate the self-reported change of loneliness, change in well-being, and change in the numbers of significant others due to Covid-19-pandemic.

**Methods:**

Computer-assisted web interviewing is being conducted. Survey design uses a multistaged sampling and iterative weighting algorithm, both based on the 2016 Hungarian micro census. The sample can be considered representative for age, gender, educational attainment, region, and size of the settlement. Continuous variables will be tested for normality of distribution using the Shapiro–Wilk, and Kolmogorov–Smirnov tests. To check the comparability of the two groups, the Mann–Whitney U tests will be applied. The cross-tabulation Pearson chi-square and Fisher’s exact tests will be performed to assess the association between categorical variables. Two-sample Z-tests will be applied to evaluate the difference between the proportions of the two groups. Multivariable logistic regression models will be also applied to understand the association between the direction of change and sociodemographic variables.

**Results:**

The survey is currently being conducted; results shall be presented at the conference.

**Conclusions:**

The survey is currently being conducted; results and conclusions shall be presented at the conference.

**Disclosure:**

No significant relationships.

